# High-Order Anharmonicities Shape Phonon Hydrodynamic Effects in Graphene

**DOI:** 10.1021/acs.nanolett.5c00855

**Published:** 2025-07-10

**Authors:** Jordi Tur-Prats, Zherui Han, Albert Beardo, Xiulin Ruan, F. Xavier Alvarez

**Affiliations:** † Departament de Física, 16719 Universitat Autònoma de Barcelona, 08193 Bellaterra, Spain; ‡ School of Mechanical Engineering and the Birck Nanotechnology Center, 6429 Purdue University, West Lafayette, Indiana 47907-2088, United States

**Keywords:** graphene, phonon hydrodynamics, four-phonon interactions, second sound, phonon viscosity

## Abstract

Prominent phonon hydrodynamic phenomena were predicted in graphene at low temperatures due to the abundance of momentum-conserving three-phonon interactions. Recent studies, however, have shown that higher-order interactions constitute an additional resistive channel that significantly reduces the thermal conductivity of this material. Here, we show that the occurrence of hydrodynamic effects in graphene is severely conditioned by four-phonon interactions. Contrary to conventional understanding, we first demonstrate that the collective limit assumption, in which the phonon distribution is fully correlated, is not adequate to understand the hydrodynamic transport mechanisms in graphene. Then we report the key hydrodynamic parameters, namely the nonlocal length and the heat flux relaxation time, and we show that they are significantly reduced if considering full anharmonicity. Finally, we discuss observable implications in a variety of experimental configurations and we critically review previous predictions on the necessary conditions for the manifestation of collective phonon behavior and phonon hydrodynamics.

## Graphical abstract

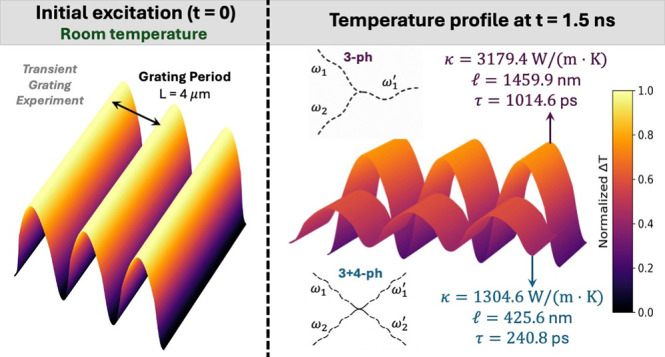

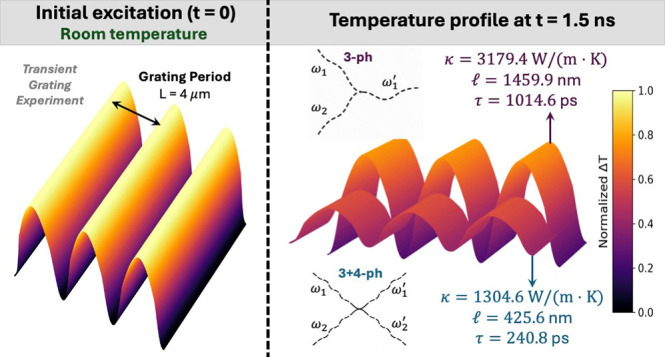

Predicting the thermal conductivity of bulk semiconductors from first-principles became possible due to the implementation of density functional theory,^1^ which informs the full landscape of phonon interactions,^2^ to solve the linearized Boltzmann Transport Equation (BTE). For simple nonequilibrium constraints, such as a homogeneous temperature gradient, the conductivity and the phonon distribution function can be obtained by solving the BTE via iterative methods^3–5^ based on the variational principle.^6^

In the presence of nanoscale boundaries, or nonhomogeneous and rapidly varying thermal fields, the phonon distribution accommodates high-order perturbations that can induce hydrodynamic-like heat transport behavior.^7^ For example, deviations from diffusion have been observed in the form of thermal waves^8,9^ and heat viscosity.^10,11^ In low-dimensional materials, these effects are amplified due to the limited phase space for phonon interactions as prescribed by conservation laws.^12–15^ Momentum-conserving phonon–phonon (Normal) collisions redistribute phonon momentum without relaxing the distribution back to equilibrium,^16^ which delays the relaxation of the heat flux and contributes to the persistence of collective phonon evolution.^17,18^ In this context, the conductivity is not the only relevant thermal property, since other integrated phonon magnitudes such as the nonlocal length and the flux relaxation time calibrate the hydrodynamic response in space and time, respectively.^7,19^

Historically, it has been assumed that accounting for three-phonon (3-ph) interactions is sufficient to fully characterize the evolution of the phonon distribution and achieve converged solutions of the BTE. Recently, however, it has been recognized that higher-order phonon scattering processes can play a non-negligible role.^20–23^ Four-phonon (4-ph) interactions introduce an additional channel of thermal resistance that profoundly influences the overall thermal transport properties in specific 2D materials such as graphene, where 3-ph scattering is restricted by a symmetry-based selection rule.^24^ Including 4-ph scattering has been shown to be crucial in converging thermal conductivity values at 300 K via the iterative method in graphene^22,25,26^ and other materials,^27,28^ while it has been shown to be negligible in other cases such as transition metal dichalcogenides.^29,30^ Thus, these higher-order anharmonicities play an important role in the context of accurate thermal management and the design of graphene-based and next-generation electronic devices, where predictive modeling of heat flow is critical. Despite their significance, the quantitative effects of four-phonon collisions on the nanoscale transport mechanisms and the hydrodynamic transport properties mentioned above remain relatively unexplored.

In this work, we discuss the origin of hydrodynamic transport effects in graphene taking into consideration the non-negligible role of 4-ph collisions, and illustrate the interplay of complex scattering mechanisms and fundamental conservation laws. Specifically, we provide a physical interpretation of the iteratively converged phonon distribution function and the associated thermal conductivity in terms of collective and kinetic contributions. We then report the key mesoscopic hydrodynamic parameters, namely the nonlocal length and the heat flux relaxation time, calculated from first-principles, and we investigate the specific role of 4-ph collisions on the emergence of viscous heat flow and second sound in a variety of experimental configurations. Overall, the analysis enables revisiting the necessary and sufficient conditions for the emergence of phonon hydrodynamics in general semiconductors at different temperatures, and questions previous interpretations.

We first consider solutions of the BTE in the presence of a constant, homogeneous temperature gradient in a system without boundaries. In such a stationary situation, the BTE can be expressed as

1 $$v_{\mu }\cdot \nabla n_{\mu }=C\left(n_{\mu }\right)$$

where *C* is the collision operator and *n*_μ_ is the phonon population of phonon mode μ = (**k**, *p*), with wavevector **k** and polarization branch *p*, and group velocity **v**_μ_.

The converged solution of eq 1 considering both 3- and 4-ph scattering processes from *ab initio* at 300 K is shown in Figure 1a (see Supporting Information for the results at 100 K). The solution is represented for acoustic branches in terms of the normalized deviation from equilibrium, and only the projection over an in-plane direction *x* is represented. We consider interatomic force constants renormalized at the corresponding temperature. More details on the iterative BTE solutions and methodology is provided in the Supporting Information.

**Figure 1:**
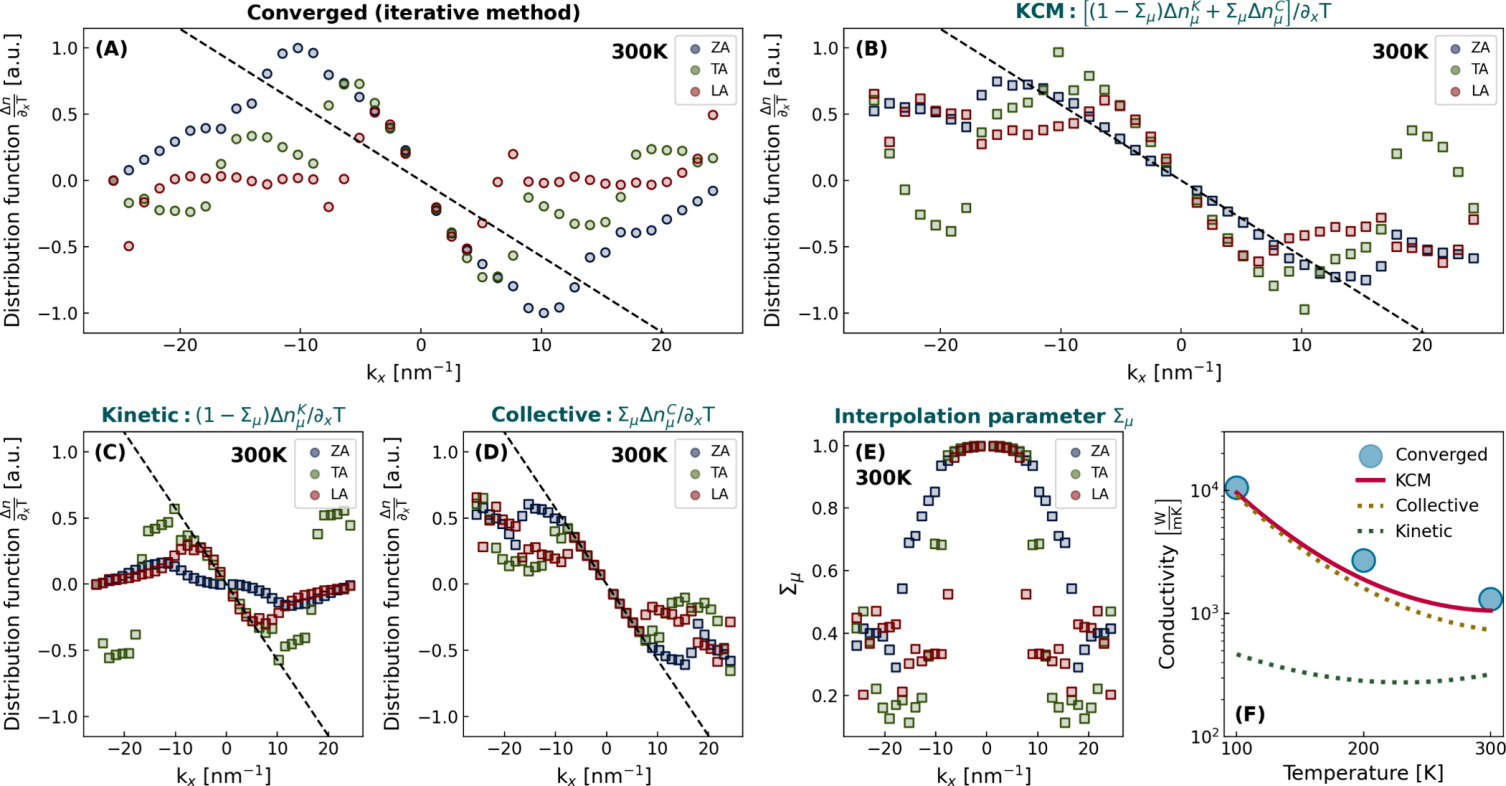
(A) Converged distribution function under a uniform thermal gradient considering 3- and 4-ph interactions at 300 K. (B) KCM distribution function in the same conditions. (C) Collective contribution. (D) Kinetic contribution. (E) Interpolation parameter. (F) Thermal conductivity at different temperatures as obtained by the iterative method and KCM, along with the collective and kinetic contributions. The displaced distribution is indicated in (A–D) as a black dashed line.

We notice that all the acoustic branches display the same slope near the zone center, which is an apparent signature of the displaced distribution associated with the collective regime (or Ziman’s limit).^6^ This characteristic has been associated with the emergence of hydrodynamic transport effects.^12,13^ However, to understand to what extent the solution in Figure 1a truly corresponds to the displaced distribution, it is necessary to distinguish kinetic and collective contributions quantifying the degree of independent and correlated phonon evolution, respectively.

We seek linearized BTE solutions in terms of the partial derivative of the equilibrium distribution *n*_μ_^0^ with respect to energy *ε* and a perturbation Φ_μ_, such that

2 $$n_{\mu }=n_{\mu }^{0}+\Delta n_{\mu }=n_{\mu }^{0}-\Phi_{\mu }\frac{dn_{\mu }^{0}}{d\varepsilon }$$

First, we consider a kinetic regime, where the conservation of momentum in phonon interactions is not frequent. In this situation, eq 1 can be simplified using the Relaxation Time Approximation (RTA)

3 $$v_{\mu }\cdot \nabla n_{\mu }=-\frac{n_{\mu }-n_{\mu }^{0}}{\tau_{\mu }^{R}}$$

where τ_μ_^*R*^ is the resistive single-mode relaxation time. In the presence of a thermal gradient (i.e., Φ_μ_ ∝ ∇*T*), we obtain

4 $$\Delta n_{\mu }^{K}=\frac{\hbar }{T}\omega_{\mu }\tau_{\mu }^{R}v_{\mu }\cdot \nabla T\frac{dn_{\mu }^{0}}{d\varepsilon }$$

where ω_μ_ is the phonon frequency. This form for *n*_μ_ suggests independent evolution of the different phonon modes and is the typical initial ansatz for the phonon distribution in iterative BTE solvers. However, as can be seen in Figure 1, the features of this solution are not reproduced by the converged solution even at 300 K.

In graphene, momentum conservation is prevalent in phonon interactions, and hence the evolution of the different modes is strongly correlated. In this case, the Collision operator cannot be simplified in terms of single-mode relaxation times, so eq 3 is not adequate. Moreover, it is easy to demonstrate that in the limit of dominant Normal collisions, or the collective regime, the solution of the BTE approaches the well-known displaced distribution,^6,31^ which is proportional to the conserved magnitude, i.e., the phonon momentum **k**:

5 $$\Delta n_{\mu }^{C}=\frac{\hbar }{T}\alpha k_{\mu }\cdot \nabla T\frac{dn_{\mu }^{0}}{d\varepsilon }$$

where α is a mode-independent constant with units of diffusivity. In contrast to the kinetic limit, the functional form of the distribution prevents independent evolution of each phonon mode, thus accommodating a collective response. To determine the collective solution, the only necessary step is the characterization of the scaling parameter α by invoking a collective constraint governing the phonon population as a whole. We impose balance of the total phonon momentum by projecting the BTE (eq 1) in terms of the mode-dependent contribution to the momentum ℏ**k**_μ_. Combining the momentum balance with eq 5 and assuming that the displaced distribution only relaxes due to resistive scattering at a rate 1/τ_μ_^*R*^, we obtain an expression for the collective diffusivity

6 $$\alpha =-\frac{\int k_{\mu }\cdot v_{\mu }\frac{dn_{\mu }^{0}}{dT}d^{2}k}{\int k_{\mu }\cdot k_{\mu }\frac{\hbar }{T\tau_{\mu }^{R}}\frac{dn_{\mu }^{0}}{d\varepsilon }d^{2}k}$$

where we neglected nonlinear terms.

Notice that eq 5 in combination with eq 6 does not correspond to a solution of the BTE under the RTA. This is expected, since eq 3 is not a correct approximation for the BTE in the collective regime. Conversely, the displaced distribution properly accounts for the complete redistribution of phonon momentum across all modes via abundant Normal scattering in between any resistive scattering events.^17^ Mathematically, this is reflected in the fact that the resistive scattering times are averaged throughout the phonon population to determine the collective perturbation in eq 5, rather than each mode being distinctly perturbed by its respective τ_μ_^*R*^.

The displaced distribution *Δn*_μ_^*C*^ is represented in Figure 1 as a black dashed line. By comparing this solution and the converged one, we observe that the slope of the acoustic branches, despite being equivalent, does not match the slope of the collective solution. This indicates that the converged solution does not correspond to the collective limit. Instead, a significant kinetic contribution is manifested, which emphasizes the key role of accounting for the full landscape of resistive scattering, including both 3- and 4-ph interactions.

To understand the significance and main physical features of the distribution, such as the slope in the zone center, it is useful to consider an interpolation between the kinetic and collective limits, as proposed by the original Kinetic-Collective Model (KCM).^17,32^ The usual interpolation considers a mode-independent parameter Σ ∈ [0, 1] that quantifies the relative importance between the momentum conserving and nonconserving relaxation times. However, in graphene, the dominance of momentum-conserving collisions is not homogeneous over the whole distribution since it is weaker away from the zone center. Therefore, here we interpolate the two limits mode by mode

7 $$\Delta n_{\mu }^{KC}=\left(1-\Sigma_{\mu }\right)\Delta n_{\mu }^{K}+\Sigma_{\mu }\Delta n_{\mu }^{C}$$

and

8 $$\Sigma_{\mu }=\frac{1}{1+\frac{\tau_{\mu }^{N}}{\tau_{\mu }^{R}}}$$

with τ_μ_^*N*^ and τ_μ_^*R*^ being the Normal and resistive scattering times, respectively. By construction, Σ → 1 in the collective limit and Σ → 0 in the kinetic one. Moreover, note that the KCM distribution, eq 7, only depends on RTA inputs.

In Figure 1, we show the KCM solution *Δn*_μ_^*KC*^, alongside the collective, Σ_μ_ *Δn*_μ_^*C*^, and kinetic, (1−Σ_μ_)*Δn*_μ_^*K*^, contributions at 300 K (see Supporting Information for results at 100 K). First, we observe that the KCM solution is much closer to the converged solution than the usual initial ansatz *Δn*_μ_^*K*^ in iterative solvers, which clearly suggests KCM as a useful method to refine the initial ansatz and reduce the required iterations for convergence and the associated computational cost. Furthermore, KCM provides a physical interpretation of the features displayed by the converged solution. On one side, the collective contribution approaches the displaced distribution only around the zone center. For wavevectors close to the edges of the Brillouin zone, Σ_μ_ < 1 and thus these modes do not remain correlated with the rest of the distribution. On the other side, the kinetic contribution of the longitudinal (LA) and transversal (TA) modes close to the zone center is very similar. This induces a deviation of the slope with respect to the collective limit, without causing a discrepancy between the slopes of these two branches. This kinetic effect explains why different branches can display the same slope without fully accommodating the collective limit. Nevertheless, the KCM predicts that this behavior is only significant for the longitudinal and transversal branches, while the converged solution indicates that the flexural (ZA) branch also deviates from the displaced distribution. This is unexpected, since the lifetimes of zone-center flexural modes are small, and hence their kinetic contribution is minor. This discrepancy might indicate that precise characterization of resistive interactions involving ZA modes is not possible by solely considering RTA inputs.

The overall consistency between the convergent solution and *Δn*_μ_^*KC*^ can also be investigated in terms of the predicted thermal conductivities. In general, from a given distribution function, the conductivity κ can be calculated by integrating the total heat flux **q**:

9 $$q=-\kappa \nabla T=\int \hbar \omega_{\mu }v_{\mu }\cdot \Delta n_{\mu }d^{2}k$$

The comparison of the thermal conductivities from the converged and KCM solutions, along with the kinetic and collective contributions, is shown in Figure 1F at different temperatures. Noteworthy, we observe that the kinetic contribution on κ is small at 300 K and negligible at lower temperatures.

Finally, this analysis emphasizes the necessity of accounting for the entire anharmonic landscape to obtain robust predictions for 2D materials. Due to the scattering selection rules in graphene,^24^ 4-ph interactions are mainly resistive and involve ZA phonons. Neglecting this form of scattering thus increases the resistive relaxation time τ_μ_^*R*^ and the kinetic deviation from equilibrium of these specific modes. However, due to the distinct role of τ_μ_^*R*^ in eqs 4 and ([Disp-formula eq5]), in the collective regime the entire phonon population is influenced by the slower relaxation of ZA phonons in the absence of 4-ph scattering, which causes a pronounced global deviation from equilibrium and a significant increase in collective diffusivity α. Furthermore, by neglecting 4-ph scattering, τ_μ_^*N*^ ≪ τ_μ_^*R*^ and Σ_μ_ ≃ 1 for all phonon modes but those very close to the edges of the Brilloiun zone. These effects combine to cause a larger increase in the amplitude of the collective contribution relative to the kinetic one in eq 7. Therefore, the KCM predicts that the distribution function closely resembles the displaced distribution if 4-ph scattering events are neglected (see Supporting Information for an extended discussion). Nevertheless, the displaced distribution is not a solution of the BTE under RTA, which is the traditional starting point for the iterative method, and it displays radically different characteristics than the kinetic solution, particularly in the zone center. Consequently, the iterative method becomes very sensitive to the discretization of the wave-vector space, which ultimately prevents numerically converging the main physical features of the distribution function and the associated thermal properties in the absence of 4-ph interactions.

Having established the role of 4-ph scattering in graphene on shaping the distribution function and the thermal conductivity under a homogeneous thermal gradient, we proceed to consider more complex nonequilibrium conditions that are prone to host fluid-like heat transport. Phonon hydrodynamics is generally manifested in the form of memory effects (second sound propagation), or viscous effects (nonlocal phonon transport).^17,33,34^ These two mechanisms are related to the slow relaxation of the heat flux compared to the characteristic time or length scales of the experimental conditions, respectively.^35^ The nonlocal length l and the flux relaxation time τ quantify the collective relaxation of the heat flux in space and time as contributed by the entire phonon population at a given temperature. The emergence of nondiffusive behavior at this specific characteristic scale is in turn a fundamental signature of hydrodynamic phonon response, in contrast to multiscale descriptions based on uncorrelated and ballistic phonon evolution.^36,37^ These quantities parametrize the simplest model accommodating hydrodynamic effects in terms of macroscopic variables such as the heat flux **q** and the temperature *T*, which is known as the Guyer-Krumhansl transport equation^7,17^

10 $$q+\tau \frac{\partial q}{\partial t}=-\kappa \nabla T+l^{2}\left(\nabla^{2}q+\zeta \nabla \nabla \cdot q\right)$$

where ζ is a dimensionless coefficient associated with volume viscosity effects. While this equation was originally predicted in the collective limit, it has been recently recognized as a general transport equation in the presence of resistive effects at moderate Knudsen numbers.^19,36,38^ In the simplest picture, the nonlocal term in eq 10 quantifies the apparent reduction of the thermal conductivity due to viscous effects at the nanoscale, and the memory term captures the wave-like or undulatory thermal response at short time scales or under high-frequency excitations. At length and time scales much larger than l and τ, respectively, eq 10 reduces to Fourier’s law of heat diffusion.

In Figure 2, we show the parameter values of the hydrodynamic equation at different temperatures as calculated from first principles. Specifically, we show the nonlocal length l , the heat flux relaxation time τ, and the volume viscosity ζ according to the simplified expressions derived in ref 7. and using the converged relaxation times quantified via the iterative method including 3- and 4-ph interactions. The required microscopic expressions are provided in the Supporting Information. This is the simplest approach to obtain an adequate estimate of the hydrodynamic parameters. However, future work may examine the parameters by iterative solving the exact expressions, also provided in ref. 7, which do not require constructing mode-dependent relaxation times from the converged distribution function.

**Figure 2:**
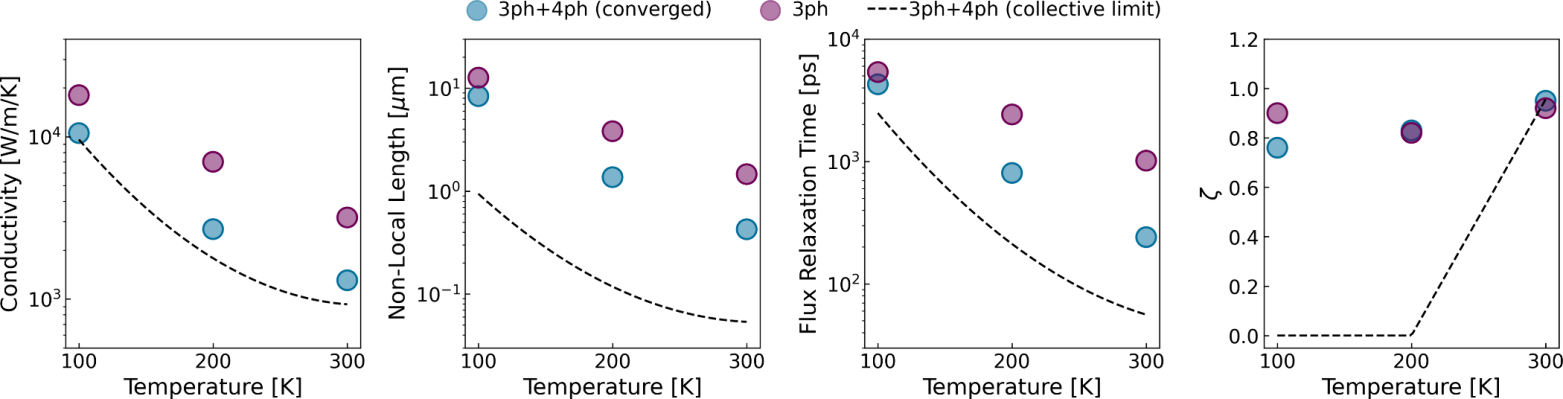
Thermal conductivity, nonlocal length, heat flux relaxation time and volume viscosity coefficient as a function of temperature. We show converged results considering 3- and 4-ph scattering rates, and the results neglecting 4-ph scattering for comparison purposes. We also show the values obtained assuming the ideal collective limit.

For comparison purposes, in Figure 2, we also show the parameter values using relaxation times resulting from iterative solutions of the BTE considering only 3-ph interactions. Interestingly, the significant effect of high-order anharmonicities on the thermal conductivity is also manifested on the hydrodynamic parameters. The reduction of the nonlocal length, for example, can be interpreted as a faster uncorrelation of the heat flux perturbations due to the additional 4-ph resistive channel. This clearly indicates that the emergence of hydrodynamic effects in graphene at the nanoscale is significantly influenced by the full anharmonic environment beyond third-order interactions, with important implications for direct microscopic approaches such as the Green’s formalism^40^ and other direct solvers of the linearized BTE.^39^ In particular, precise sampling of low-frequency phonons is crucial, since they display a dominant contribution on the converged value of l and τ whereas displaying a relatively smaller contribution on κ.

As a reference, in Figure 2 we also show the values of the hydrodynamic parameters using the original expressions proposed by Guyer-Krumhansl.^17^ These results correspond to an ideal approximation only valid in the Ziman’s limit, τ_μ_^*N*^ ≪ τ_μ_^*R*^.^15^ Importantly, even though the shape of the distribution function and the thermal conductivity have a dominant collective contribution, we show that the microscopic expressions derived particularly for the collective regime underestimate the hydrodynamic properties of graphene even at 100 K. Therefore, we conclude that the collective limit is neither an adequate assumption to predict phonon hydrodynamic effects nor a necessary condition for the presence of hydrodynamic heat transport.

The *ab initio* parametrization of eq 10 allows us to simulate paradigmatic experimental configurations displaying strong nondiffusive behavior. In Figure 3, we show the thermal response predicted under ring-shaped,^39^ and grating^8,41^ optical excitations along with predictions of steady-state heat flow in nanoribbons.^11^ Since these particular experimental conditions have been investigated in graphite samples, they represent an adequate testbed to illustrate hydrodynamic phenomenology potentially measurable in monolayer graphene. Using Finite Elements,^42^ we simulate the different configurations by combining the hydrodynamic heat transport eq 10 with the energy balance equation, *C*_*v*_∂_*t*_ *T* = −∇·**q** + *Q*, with *C*_*v*_ being the volumetric specific heat capacity, and *Q* the external power density sources. In the nanoribbons, we consider slip boundary conditions assuming fully diffusive phonon-boundary scattering.^42^ We use the *ab initio* calculated parameters reported in Figure 2 for the different predictions.

**Figure 3:**
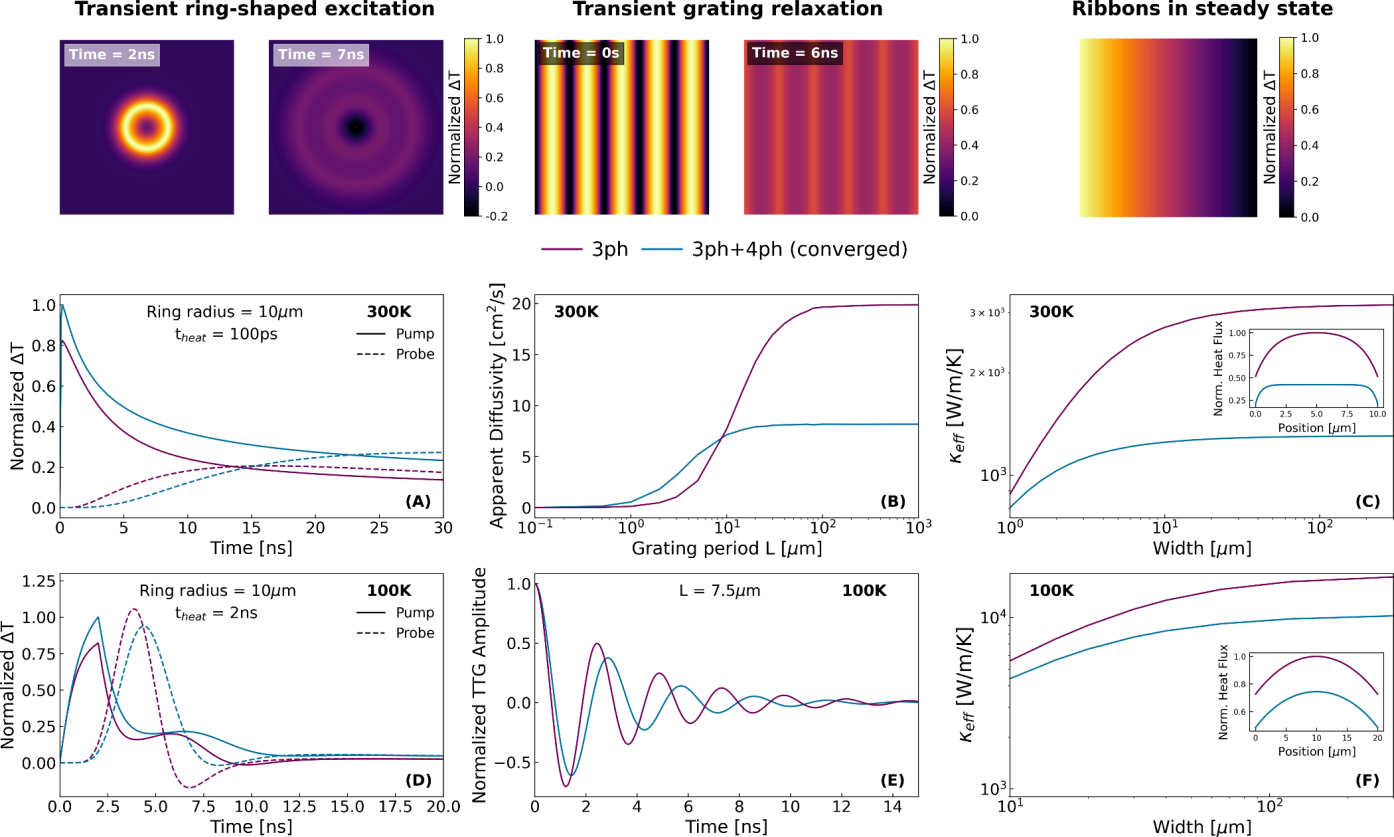
Predictions of the nanoscale thermal response in different configurations considering 3-ph and 3ph+4ph interactions at 300 and 100 K. (A,D) Thermal relaxation in response to a ring-shaped optical excitation.^39^ The evolution of the normalized temperature is shown in the excitation region (pump) and in the center of the ring (probe). The radius of the ring and the duration of the heating time-window t_*heat*_ is indicated in each case. (B,E) Thermal relaxation in response to grating optical excitation with grating period *L*. At 300 K, the apparent diffusivity as a function of the grating period is shown. At 100 K, the time-evolution of the transient grating amplitude for *L* = 7.5 μm identifies second sound propagation. (C,F) Apparent steady-state conductivity in nanoribbons as a function of the width. The insets display the heat flux transversal profile for a width of 10 and 20 μm at 300 and 100 K, respectively.

At 300 K, the memory term has a negligible effect on the solutions, so the deviations from diffusion emerge as a viscous reduction of the apparent thermal conductivity (or apparent diffusivity) depending on the optical ring size, the transient grating period, or the nanoribbon width, respectively, as shown in Figure 3. When the experimental length scale becomes comparable to the nonlocal length, the heat flux Laplacian term in eq 10 becomes significant, which reduces the amount of energy that flows for a given thermal gradient and delays the homogenization of the temperature profile. In this case, using converged parameters accounting for 3- and 4-ph collisions causes a radical deviation on the predictions using only 3-ph. First, the conductivity/diffusivity in the macroscopic limit (bulk) is reduced by a factor of ∼3 (cf. Figure 3B), which explains the slower relaxation of the ring-shaped excitation at 300 K in Figure 3A. In addition, the reduction of the nonlocal length by 4-ph scattering causes the nanoscale viscous effects to emerge at smaller transient grating periods or nanoribbon widths. In particular, the results neglecting 4-ph scattering predict deviations from bulk diffusive behavior at grating periods *L* < 100 μm, whereas the refined calculation predicts that nondiffusive behavior emerges at significantly smaller length scales, *L* < 20 μm. This is also reflected in the stationary heat flux profiles established in the nanoribbons, where the role of 4-ph scattering restricts the viscous response to a smaller boundary layer,^42^ thus flattening the profile.

At 100 K, the nonlocal length predicted from *ab initio* becomes much larger than the typical grating periods or ring sizes currently achievable in experiments.^8,39^ In this situation, the spatial heat flux correlations become geometrically constrained. To model this effect, simple geometrical expressions can be used to constrain the value of l in terms of the smallest experimental length scale.^10^ For transient grating experiments with period *L*, we propose l=L/6 , motivated by the relevant length scale in these experiments,^43^ and for ring-shaped excitations with radius *R*, we propose l=R/4 . We use *ab initio* values for the other parameters. Accordingly, the viscous response diminishes and the memory effect dominates in the present transient experiments at 100 K, which unlocks second sound propagation. In Figure 3, the undulatory behavior of the thermal signals, such as the observed cooling in the center of the ring, indicates that a fraction of the thermal energy propagates as a wave.^8,39^ However, due to the reduction of the heat flux relaxation time τ in the presence of 4-ph scattering (cf. Figure 2), we predict a lessening of the undulatory behavior in both experiments, which manifests as a reduced amplitude and velocity of the thermal wave. This hydrodynamic attenuation is also predicted at higher temperatures, thus limiting the experimental window for the observation of second sound at room temperature to extremely small grating periods or ring sizes and reducing the fraction of thermal energy propagating as a wave relative to the diffusive background. Nonetheless, recent TG experiments using short wavelength optical pulses have demonstrated grating excitations of 10s and 100s of nanometers,^44,45^ which is promising for investigating wavelike deviations from thermal diffusion at high temperatures.

In conclusion, the full phonon–phonon scattering environment, including 4-ph interactions, must be considered to predict collective and hydrodynamic behavior in graphene at temperatures ranging from 100 to 300 K. Here we have interpreted the complex interplay between momentum-conserving and resistive interactions in terms of an interpolated BTE solution between the collective and kinetic limits, which in turn provides a refined initial ansatz for the distribution function in iterative BTE solvers. We have also shown that the Guyer-Krumhansl equation compactly predicts the distinct hydrodynamic heat transport effects at the nanoscale in terms of intrinsic material properties that can be calculated from first-principles considering full anharmonicity. Primarily, we have demonstrated that the additional resistive relaxation channel induced by 4-ph scattering not only causes a reduction of the thermal conductivity, but also diminishes the nonlocal length and heat flux relaxation time. This causes a significant attenuation of the phonon hydrodynamic phenomena emerging at the nanoscale and narrows the experimental window for their observation.

## Supplementary Material



## Notes

Microscopic expressions required to calculate the hydrodynamic thermal transport parameters, details on the numerical iterative solver of the BTE used to calculate converged relaxation times, and extended discussion of the role of 4-ph scattering in graphene along with KCM solutions neglecting 4-ph scattering (PDF)

## References

[1] Debernardi A., Baroni S., Molinari E. (1995). Anharmonic Phonon Lifetimes in Semiconductors
from Density-Functional Perturbation Theory. Phys. Rev. Lett..

[2] Lindsay L., Hua C., Ruan X.L., Lee S. (2018). Survey of
ab initio phonon thermal
transport. Materials Today Physics.

[3] Broido D. A., Malorny M., Birner G., Mingo N., Stewart D. A. (2007). Intrinsic
lattice thermal conductivity of semiconductors from first principles. Appl. Phys. Lett..

[4] Li W., Carrete J., Katcho N. A., Mingo N. (2014). ShengBTE: a solver
of the Boltzmann transport equation for phonons. Comput. Phys. Commun..

[5] Omini M., Sparavigna A. (1995). An iterative approach to the phonon
Boltzmann equation
in the theory of thermal conductivity. Physica
B: Condensed Matter.

[6] Ziman, J. M. Electrons and Phonons: The Theory of Transport Phenomena in Solids; Clarendon Press: 2001.

[7] Sendra L., Beardo A., Torres P., Bafaluy J., Alvarez F. X., Camacho J. (2021). Derivation of a hydrodynamic heat equation from the
phonon Boltzmann equation for general semiconductors. Phys. Rev. B.

[8] Huberman S., Duncan R. A., Chen K., Song B., Chiloyan V., Ding Z., Maznev A. A., Chen G., Nelson K. A. (2019). Observation
of second sound in graphite at temperatures above 100 K. Science.

[9] Beardo A., López-Suárez M., Pérez L. A., Sendra L., Alonso M. I., Melis C., Bafaluy J., Camacho J., Colombo L., Rurali R. (2021). Observation
of second sound in a rapidly varying temperature field in Ge. Science advances.

[10] Beardo A., Knobloch J. L., Sendra L., Bafaluy J., Frazer T. D., Chao W., Hernandez-Charpak J.
N., Kapteyn H. C., Abad B., Murnane M. M., Alvarez F. X., Camacho J. (2021). A General
and Predictive Understanding of Thermal Transport from 1D- and 2D-Confined
Nanostructures: Theory and Experiment. ACS Nano.

[11] Huang X., Guo Y., Wu Y., Masubuchi S., Watanabe K., Taniguchi T., Zhang Z., Volz S., Machida T., Nomura M. (2023). Observation
of phonon Poiseuille flow in isotopically purified graphite ribbons. Nat. Commun..

[12] Lee S., Broido D., Esfarjani K., Chen G. (2015). Hydrodynamic phonon
transport in suspended graphene. Nat. Commun..

[13] Cepellotti A., Fugallo G., Paulatto L., Lazzeri M., Mauri F., Marzari N. (2015). Phonon hydrodynamics
in two-dimensional materials. Nat. Commun..

[14] Shang M.-Y., Mao W.-H., Yang N., Li B., Lü J.-T. (2022). Unified
theory of second sound in two-dimensional materials. Phys. Rev. B.

[15] Sendra L., Beardo A., Bafaluy J., Torres P., Alvarez F. X., Camacho J. (2022). Hydrodynamic heat transport in dielectric crystals
in the collective limit and the drifting/driftless velocity conundrum. Phys. Rev. B.

[16] Callaway J. (1959). Model for
Lattice Thermal Conductivity at Low Temperatures. Phys. Rev..

[17] Guyer R. A., Krumhansl J. A. (1966). Solution of the Linearized Phonon Boltzmann Equation. Phys. Rev..

[18] Hardy R. J. (1970). Phonon
Boltzmann equation and second sound in solids. Phys. Rev. B.

[19] Guo Y., Wang M. (2018). Phonon hydrodynamics
for nanoscale heat transport at ordinary temperatures. Phys. Rev. B.

[20] Feng T., Ruan X. (2016). Quantum mechanical prediction of four-phonon scattering rates and
reduced thermal conductivity of solids. Phys.
Rev. B.

[21] Feng T., Lindsay L., Ruan X. (2017). Four-phonon
scattering significantly
reduces intrinsic thermal conductivity of solids. Phys. Rev. B.

[22] Feng T., Ruan X. (2018). Four-phonon scattering
reduces intrinsic thermal conductivity of
graphene and the contributions from flexural phonons. Phys. Rev. B.

[23] Xia Y. (2018). Revisiting
lattice thermal transport in PbTe: The crucial role of quartic anharmonicity. Appl. Phys. Lett..

[24] Lindsay L., Broido D., Mingo N. (2010). Flexural phonons
and thermal transport
in graphene. Phys. Rev. B.

[25] Han Z., Ruan X. (2023). Thermal conductivity of monolayer graphene: Convergent
and lower
than diamond. Phys. Rev. B.

[26] Tong Z., Pecchia A., Yam C., Dumitrică T., Frauenheim T. (2022). Ultrahigh Electron Thermal Conductivity in T-Graphene,
Biphenylene, and Net-Graphene. Adv. Energy Mater..

[27] Guo Z., Han Z., Alkandari A., Khot K., Ruan X. (2024). First-principles prediction
of thermal conductivity of bulk hexagonal boron nitride. Appl. Phys. Lett..

[28] Sun G., Ma J., Liu C., Xiang Z., Xu D., Liu T.-H., Luo X. (2023). Four-phonon
and normal scattering in 2D hexagonal structures. Int. J. Heat Mass Transfer.

[29] Han Z., Sokalski P., Shi L., Ruan X. (2023). Prediction of hot zone-center
optical phonons in laser-irradiated molybdenum disulfide with a semiconductor
multitemperature model. Phys. Rev. B.

[30] Farris R., Hellman O., Zanolli Z., Saleta Reig D., Varghese S., Ordejón P., Tielrooij K.-J., Verstraete M. J. (2024). Microscopic understanding of the
in-plane thermal transport
properties of 2*H* transition metal dichalcogenides. Phys. Rev. B.

[31] Sussmann J., Thellung A. (1963). Thermal conductivity of perfect dielectric crystals
in the absence of umklapp processes. Proceedings
of the Physical Society.

[32] Torres P., Torelló A., Bafaluy J., Camacho J., Cartoixà X., Alvarez F. X. (2017). First principles kinetic-collective thermal conductivity
of semiconductors. Phys. Rev. B.

[33] Alvarez F. X., Jou D. (2007). Memory and nonlocal
effects in heat transport: From diffusive to
ballistic regimes. Appl. Phys. Lett..

[34] Luo X.-P., Guo Y.-Y., Yi H.-L. (2024). Nonlocal
phonon thermal transport
in graphene in hydrodynamic regime. J. Phys.:
Condens. Matter.

[35] Hardy R. J. (1963). Energy-Flux
Operator for a Lattice. Phys. Rev..

[36] Beardo A., Alajlouni S., Sendra L., Bafaluy J., Ziabari A., Xuan Y., Camacho J., Shakouri A., Alvarez F. X. (2022). Hydrodynamic
thermal transport in silicon at temperatures ranging from 100 to 300
K. Phys. Rev. B.

[37] Raya-Moreno M., Carrete J., Cartoixà X. (2022). Hydrodynamic
signatures in thermal
transport in devices based on two-dimensional materials: An ab initio
study. Phys. Rev. B.

[38] Xiang Z., Jiang P., Yang R. (2022). Time-domain thermoreflectance
(TDTR)
data analysis using phonon hydrodynamic model. J. Appl. Phys..

[39] Jeong J., Li X., Lee S., Shi L., Wang Y. (2021). Transient Hydrodynamic
Lattice Cooling by Picosecond Laser Irradiation of Graphite. Phys. Rev. Lett..

[40] Chiloyan V., Huberman S., Ding Z., Mendoza J., Maznev A. A., Nelson K. A., Chen G. (2021). Green’s functions of the Boltzmann
transport equation with the full scattering matrix for phonon nanoscale
transport beyond the relaxation-time approximation. Phys. Rev. B.

[41] Ding Z., Chen K., Song B., Shin J., Maznev A. A., Nelson K. A., Chen G. (2022). Observation of second sound in graphite
over 200 K. Nat. Commun..

[42] Beardo A., Calvo-Schwarzwälder M., Camacho J., Myers T., Torres P., Sendra L., Alvarez F., Bafaluy J. (2019). Hydrodynamic
Heat Transport in Compact and Holey Silicon Thin Films. Phys. Rev. Appl..

[43] Maznev A. A., Johnson J. A., Nelson K. A. (2011). Onset of nondiffusive phonon transport
in transient thermal grating decay. Phys. Rev.
B.

[44] Mincigrucci R., Capotondi F., Foglia L., Naumenko D., Maznev A., Pedersoli E., Simoncig A., Caporaletti F. (2019). Nanoscale transient
gratings excited and probed by extreme ultraviolet
femtosecond pulses. Science advances.

[45] Nelson E. E., McBennett B., Culman T. H., Beardo A., Kapteyn H. C., Frey M. H., Atkinson M. R., Murnane M. M., Knobloch J. L. (2024). Tabletop
deep-ultraviolet transient grating for ultrafast nanoscale carrier-transport
measurements in ultrawide-band-gap materials. Physical Review Applied.

