# Supplementary material for: High-Order Anharmonicities Shape Phonon Hydrodynamic Effects in Graphene
Source: Nano Lett. 2025 Jul 10;25(29):11203–9. doi: 10.1021/acs.nanolett.5c00855 (PMC12291587; doi:10.1021/acs.nanolett.5c00855)
Supplement: Supplementary file 1 [file nl5c00855_si_001.pdf]

SUPPLEMENTARY MATERIAL: High-order anharmonicities shape phonon hydrodynamic effects in graphene

Jordi Tur-Prats,[1] Zherui Han,[2] Albert Beardo,[1,*] Xiulin Ruan,[2] and F. X. Alvarez[1]

[1]*Departament de Física, Universitat Autònoma de Barcelona, 08193 Bellaterra, Catalonia, Spain*
[2]*School of Mechanical Engineering and the Birck Nanotechnology Center, Purdue University, West Lafayette, Indiana 47907-2088, USA*

I. HYDRODYNAMIC PARAMETERS EXPRESSIONS

Here we show all the expressions used to obtain the *ab initio* values of the hydrodynamic parameters. The derivation of the hydrodynamic equation from the BTE along with the general expressions for the parameters can be found in Ref. [1]. Here we use the simplified experessions, also provided in Ref. [1], to evaluate the parameters in terms of the relaxation times τ_μ characterized from the iteratively converged solution of the BTE [2, 3]. Using brackets to denote a weighted average by the specific heat, $\langle x_\mu \rangle \equiv \int \hbar\omega_\mu \partial_T n^0_\mu x_\mu d\mu / C_v$, the expressions read,

$$\kappa = \frac{1}{d} C_v \langle v_\mu^2 \tau_\mu \rangle, \tag{1}$$

$$\tau = \frac{\langle v_\mu^2 \tau_\mu^2 \rangle}{\langle v_\mu^2 \tau_\mu \rangle}, \tag{2}$$

$$\ell^2 = \frac{1}{d+2} \frac{\left\langle \frac{v_\mu^3}{v_{p,\mu}} \tau_\mu^2 \right\rangle}{\left\langle \frac{v_\mu}{v_{p,\mu}} \right\rangle}, \tag{3}$$

$$\tau^e = \tau - \langle \tau_\mu \rangle, \tag{4}$$

$$\zeta = 2 - \frac{d+2}{d} \frac{\langle v_\mu^2 \tau_\mu \rangle \left\langle \frac{v_\mu}{v_{p,\mu}} \tau_\mu \right\rangle}{\left\langle \frac{v_\mu^3}{v_{p,\mu}} \tau_\mu^2 \right\rangle} - \frac{\kappa \tau^e}{C_v \ell^2}, \tag{5}$$

where v_p is the phase velocity, and $d = 2$ is the dimensionality.

Collective limit

Assuming the ideal collective limit ($\tau_{N,\mu} \ll \tau_{R,\mu}$), the general expressions reduce to the following [4],

$$\kappa_C = \frac{1}{d} C_v \left\langle \frac{v_\mu}{v_{p,\mu}} \right\rangle^2 \langle v_{p,\mu}^{-2} \rangle^{-1} \tau_C \tag{6}$$

$$\ell_C^2 = \frac{1}{d+2} \left\langle \frac{v_\mu^2}{v_{p,\mu}^2} \tau_{N,\mu} \right\rangle \langle v_{p,\mu}^{-2} \rangle^{-1} \tau_C, \tag{7}$$

* albert.beardo@uab.cat

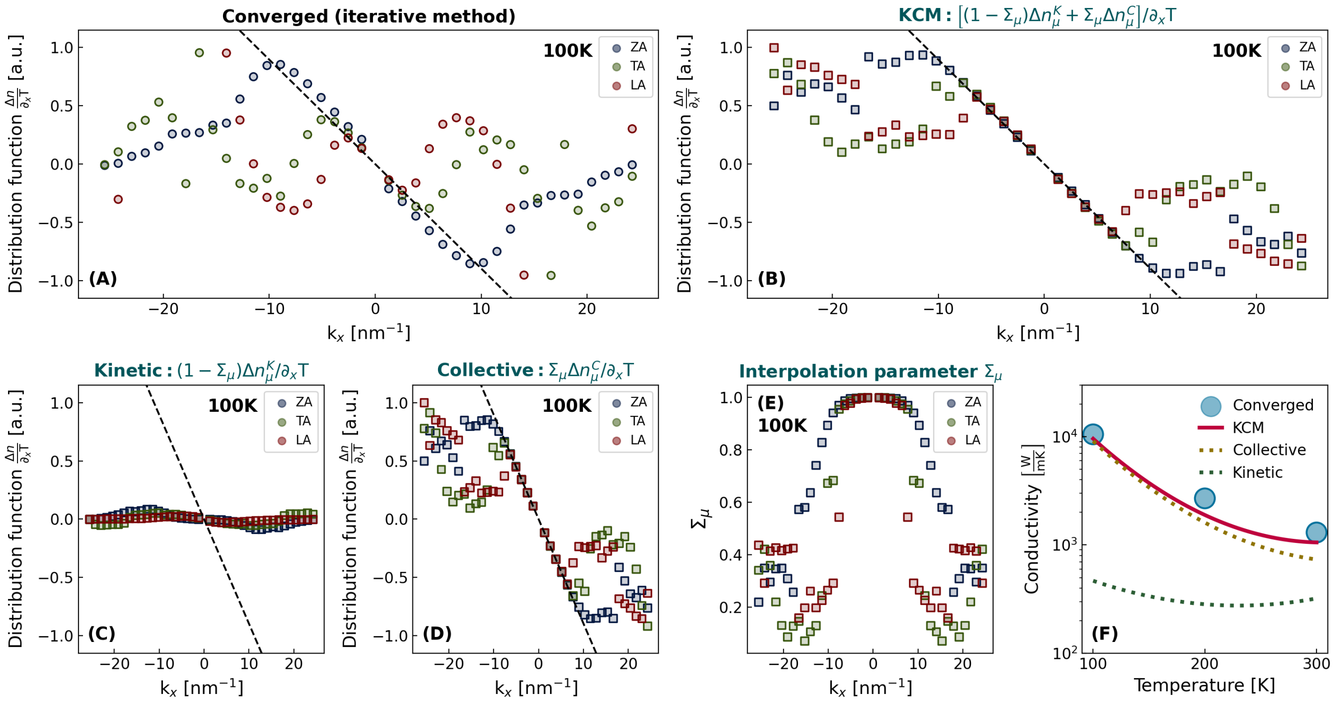

FIG. 1. **(A)** Converged distribution function under a uniform thermal gradient considering 3- and 4-ph interactions at 100 K. **(B)** KCM distribution function in the same conditions. **(C)** Collective contribution. **(D)** Kinetic contribution. **(E)** Interpolation parameter. **(F)** Thermal conductivity at different temperatures as obtained by the iterative method and KCM, along with the collective and kinetic contributions. The displaced distribution is indicated in **(A-D)** as a black dashed line.

$$\tau_C^e = \left\langle \frac{v_\mu}{v_{p,\mu}} \tau_{N,\mu} \right\rangle \left\langle \frac{v_\mu}{v_{p,\mu}} \right\rangle - \langle \tau_{N,\mu} \rangle, \tag{8}$$

$$\zeta_C = 2 - \frac{d+2}{d} \frac{\left\langle \frac{v}{v_{p,\mu}} \tau_{N,\mu} \right\rangle}{\left\langle \frac{v_\mu^2}{v_{p,\mu}^2} \tau_{N,\mu} \right\rangle} \left\langle \frac{v_\mu}{v_{p,\mu}} \right\rangle - \frac{\kappa_C \tau_C^e}{C_v \ell_C^2}, \tag{9}$$

where τ_C is the collective relaxation time directly calculated from the displaced distribution function as shown in Ref. [4]. We note that expression (6) is compatible with the collective contribution to the conductivity in the KCM formulation derived in the main text. Most importantly, the expressions derived in the collective limit underestimate the hydrodynamic parameters even at low temperatures (cf. Figure 2 of the main text).

II. ITERATIVE SOLUTIONS OF THE BTE

To iteratively solve the linearized BTE and obtain converged relaxation times τ_μ, we follow the methodology discussed in Ref. [5]. For solutions considering both 3-ph and 4-ph interactions, we consider a mesh of 40×40×1 q-points. This is sufficient to obtain converged results for the thermal conductivity in graphene [6]. For solutions considering only 3-ph interactions, we consider a finer mesh of 80×80×1 q-points to approach a result less sensitive to the exact discretization in wave-vector space. At 100 K, we impose a limiting length scale of 10 μm for the converged value of the mean free paths to facilitate convergence. For all the calculations we use interatomic force constants renormalized at each corresponding temperature [6].

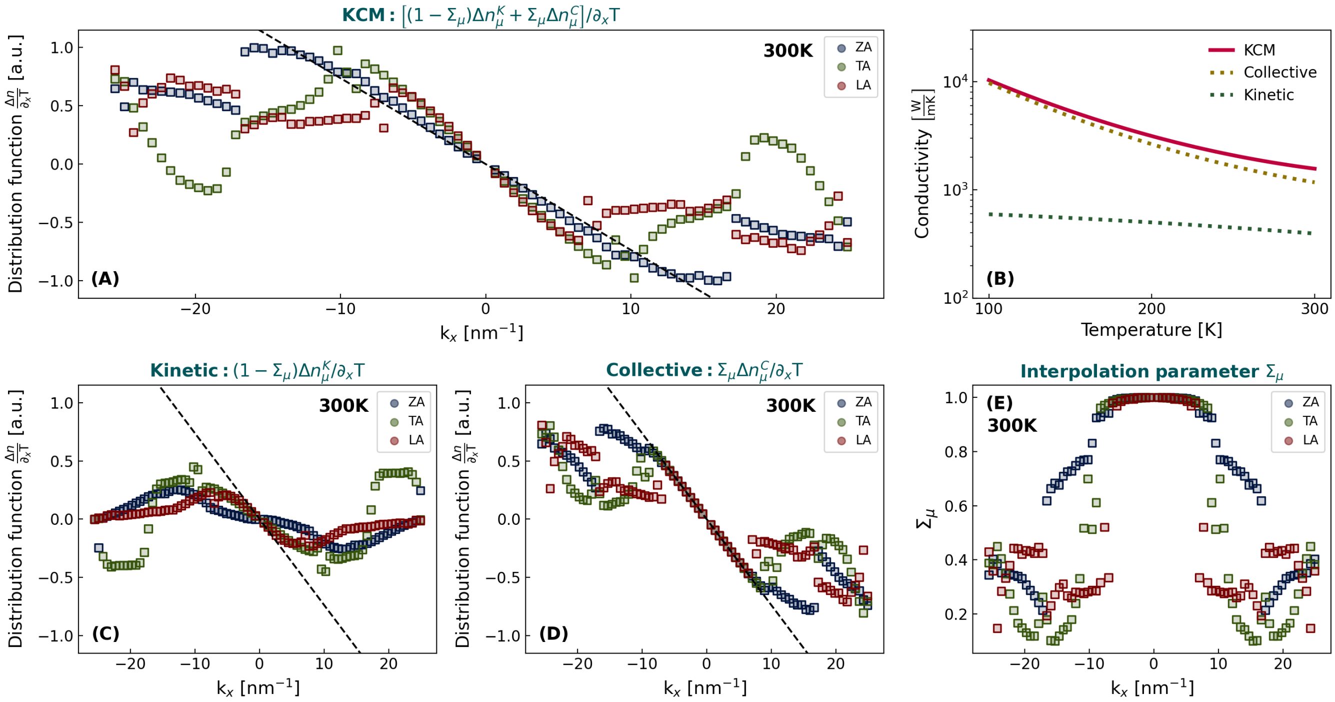

FIG. 2. (A) KCM distribution function under a uniform thermal gradient considering only 3-ph interactions at 300 K. (B) Thermal conductivity at different temperatures as obtained by KCM, along with the collective and kinetic contributions. (C) Kinetic contribution. (D) Collective contribution. (E) Interpolation parameter. The displaced distribution is indicated in (A,C,D) as a black dashed line.

III. EXTENDED DISCUSSION ON THE INFLUENCE OF 4-PH SCATTERING

As already anticipated in previous work [6], the main effect of 4-ph scattering is reducing the resistive scattering time of low-frequency ZA modes. The kinetic deviation from equilibrium, which scales proportionally with τ_μ^R, is thus mainly modified for this specific fraction of the phonon spectrum. Conversely, in the collective regime, Normal scattering simultaneously and synchronously relaxes all modes by rapidly transferring momentum and repopulating the modes that interact more frequently via resistive scattering [7]. Hence, these additional relaxation channels modify the deviation from equilibrium of the entire phonon population, including the high-frequency portion of the phonon spectrum that interacts infrequently via 4-ph scattering. Consistently, the collective perturbation described by Eqs. (5) and (6) of the main text is a function of a collective (mode-independent) relaxation time that is obtained by averaging the inverse of τ_R in the collective diffusivity α.

As a consequence of the different effect of modifying the scattering times in each limit, the kinetic contribution to the conductivity increases by 23.1% by neglecting 4-ph interactions at 300 K, while the collective conductivity contribution increases by 59.9%. As shown in Figure 2, this also causes the collective part of the distribution to dominate over the kinetic one, which ultimately causes a stronger resemblance between the KCM distribution function and the displaced distribution in the absence of 4-ph scattering [8]. In conclusion, high-order interactions represent an important factor underlying the significant kinetic contribution identified in graphene at 300 K (see Figure 1 of the main text).

[1] L. Sendra, A. Beardo, P. Torres, J. Bafaluy, F. X. Alvarez, and J. Camacho, Derivation of a hydrodynamic heat equation from the phonon boltzmann equation for general semiconductors, Phys. Rev. B **103**, L140301 (2021).

[2] T. Feng, L. Lindsay, and X. Ruan, Four-phonon scattering significantly reduces intrinsic thermal conductivity of solids, Phys. Rev. B **96**, 161201 (2017).

[3] T. Feng and X. Ruan, Quantum mechanical prediction of four-phonon scattering rates and reduced thermal conductivity of solids, Phys. Rev. B **93**, 045202 (2016).

[4] L. Sendra, A. Beardo, J. Bafaluy, P. Torres, F. X. Alvarez, and J. Camacho, Hydrodynamic heat transport in dielectric

crystals in the collective limit and the drifting/driftless velocity conundrum, Phys. Rev. B **106**, 155301 (2022).

[5] Z. Han, X. Yang, W. Li, T. Feng, and X. Ruan, Fourphonon: An extension module to shengbte for computing four-phonon scattering rates and thermal conductivity, Computer Physics Communications **270**, 108179 (2022).

[6] Z. Han and X. Ruan, Thermal conductivity of monolayer graphene: Convergent and lower than diamond, Phys. Rev. B **108**, L121412 (2023).

[7] R. A. Guyer and J. A. Krumhansl, Solution of the linearized phonon boltzmann equation, Phys. Rev. **148**, 766 (1966).

[8] S. Lee, D. Broido, K. Esfarjani, and G. Chen, Hydrodynamic phonon transport in suspended graphene, Nature communications **6**, 6290 (2015).
